# Feasibility demonstration of a device for vitreous liquid biopsy incidental to intravitreal injection

**DOI:** 10.1371/journal.pone.0294526

**Published:** 2024-01-19

**Authors:** Alexandre R. Tumlinson, Jennifer M. Calara, Dimitri T. Azar, Anthony P. Adamis, Demetrios G. Vavvas, Jay M. Stewart

**Affiliations:** 1 Twenty / Twenty Therapeutics LLC, South San Francisco, California, United States of America; 2 Department of Ophthalmology, Mass Eye and Ear, Harvard Medical School, Boston, Massachusetts, United States of America; 3 Department of Ophthalmology, University of California, San Francisco, San Francisco, California, United States of America; 4 Department of Ophthalmology, Zuckerberg San Francisco General Hospital and Trauma Center, San Francisco, California, United States of America; University of Rochester FEI: University of Rochester David and Ilene Flaum Eye Institute, UNITED STATES

## Abstract

**Purpose:**

VitreoDx is an experimental device enabling push-button collection of a neat vitreous liquid biopsy incidental to an intravitreal injection. We explored the ability of the device to collect a sample usable for proteomic biomarker discovery and testing.

**Design:**

Pilot study using *ex vivo* human eyes.

**Methods:**

Non-vitrectomized, human eyes from nine donors 75–91 years of age were refrigerated in BSS and used within 5 days of death. Four VitreoDx devices fitted with 25G needles, and four staked needle insulin syringes with 30G needles, were inserted at equal intervals through the pars plana of each eye and held in place by a fixture. The sampling mode of each VitreoDx device was triggered to attempt to acquire a liquid biopsy up to 70 μL. The plunger of each insulin syringe was retracted to attempt to obtain a liquid biopsy with a maximum volume of 50 μL. Samples acquired with the VitreoDx were extracted to polypropylene cryovials, refrigerated to -80 ºC, and sent for offsite proteomic analysis by proximity extension assay with a focus on panels containing approved and pipelined drug targets for neovascular disease and inflammatory factors.

**Results:**

Of the attempted liquid biopsies with the novel 25G VitreoDx, 92% (66 of 72) resulted in successful acquisition (>25 μL) while 89% (64 of 72) attempted by a traditional 30G needle resulted in a successful acquisition. Sample volume sufficient for proteomics array analysis was acquired by the VitreoDx for every eye. Detectable protein was found for 151 of 166 unique proteins assayed in at least 25% of eyes sampled by VitreoDx.

**Conclusions:**

The high acquisition rate achieved by the prototype was similar to that achieved in previous clinical studies where a standard syringe was used with a 25G needle to biopsy vitreous fluid directly prior to standard intravitreal injection. Successful aspiration rates were likewise high for 30G needles. Together, these suggest that it is possible to routinely acquire liquid vitreous biopsies from patients who typically receive intravitreal injections with an injection device using a standard size needle without a vitreous cutter. Protein analysis shows that proteins of interest survive the sampling mechanism and may have potential to direct care in the future.

## Introduction

The vitreous humor’s proximity to the retina, and its relative metabolic inactivity, render it an effective depot in which to inject drugs to modulate retinal disease or from which retinal disease-relevant proteins, with possible diagnostic significance, can be measured [1]. In studies of both aqueous and vitreous humor acquired from the same individual, the concentration of multiple protein biomarkers has been shown to somewhat loosely correlated, suggesting that vitreous protein concentration may be more actionable than aqueous for guiding retinal care decisions [2]. Although most studies analyzing vitreous humor use samples collected during surgical vitrectomy, reports show that vitreous fluid can be safely and reliably aspirated as a liquid biopsy, in-office, with a 25G needle directly prior to a normally indicated intravitreal injection [3, 4]. Despite the safety record demonstrated with 25G needle tap under these circumstances, researchers remain hesitant to penetrate the posterior chamber without a demonstrated clinical benefit, and typically perform this operation only when a sample is immediately required to establish a vitreous culture, as in cases of endophthalmitis or uveitis [5]. For research applications, it is essential that large libraries of samples are collected with minimal bias, particularly as the number of simultaneously investigated proteins increases [6]. In order to translate a vitreous biomarker test to the clinic and achieve the promise of routine in-office vitreous biopsy for individuals receiving intravitreal injections, it is vital to introduce a method that can reliably acquire a usable sample while further minimizing additional trauma, both psychological and physical, to the patient. To these ends, we have developed VitreoDx: a device simplifying collection of a vitreous liquid biopsy, of pre-specified volume, unpolluted by the injected drug, as a part of the intravitreal injection process. This study tests the ability of a prototype VitreoDx implementation to acquire liquid biopsies from *ex vivo* human eyes and demonstrates the use of modern panel assays to analyze those samples for proteins of interest.

## Materials and methods

**Fig 1:**
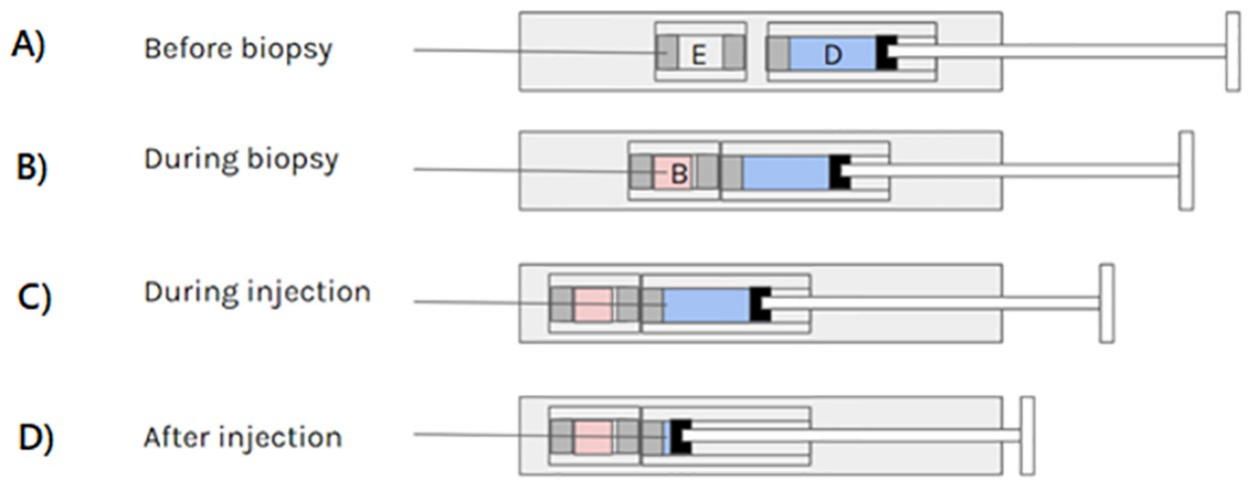
Principal and operation of VitreoDx device. In its initial state A), the double ended needle fixed into the outer housing has an interior septum piercing end which is buried in the distal septum of the evacuated sample container ‘E’. A drug cartridge ‘D’ with a distal septum and a plunger sits proximally adjacent to the sample chamber. Depressing the plunger to a first stop transitions the device to an aspiration state B) by advancing the cartridge and the sample chamber such that the needle pierces the sample chamber and applies vacuum to needle, thereby aspirating a liquid biopsy ‘B’. Further depressing the plunger moves the device into a injection state C), advancing the cartridge and the sample chamber until the needle pierces the drug cartridge. At this point, any further depression of the plunger acts to force any drug contained in the cartridge out of the needle until the device reaches a completed state D). Note that drug cartridge was, for the experiment described here, empty to avoid introducing non-vitreous fluid to the eye.

Prototype VitreoDx devices were constructed which were capable of first acquiring a neat liquid biopsy, followed by ejecting a volume of drug through the same needle. The device can be described as a cartridge injector with an intermediate pierceable vacuum sample chamber (Fig 1). Its architecture included a double ended needle, a pierceable drug cartridge, and a novel pierceable evacuated sample chamber. The double-ended needle was fixed at the distal end of a barrel in which the pierceable evacuated chamber was inserted, followed by the pierceable drug cartridge. The interior end of the double-ended needle was initially embedded in an elastic sealing layer on the surface of the evacuated chamber. The prototypes used in this experiment were operated with an empty drug cartridge. The mass of each device was recorded prior to use with a precision balance (Sartorius, Göttingen, Germany).

Whole-globe, *ex vivo* human eyes were acquired from Eversight Center for Vison and Eye Banking Research (Cleveland, OH). Tissues were collected between June and August 2022. Uniform Anatomical Gift Act authorization to use the tissue for research was obtained for each donated tissue [7]. Incomplete medical and ophthalmic history was available for some but not all eyes (Table 1) (Sheet ‘demographics and aspiration success’ in S1 Appendix). Enucleated eyes were refrigerated and shipped in BSS. All eyes appeared undeformed and firm to digital pressure at time of use.

**Table 1:** Donor demographics and medical history.

Age Mean ± SD (range)	78.8 ± 4.9 (75–91) years	
Male:Female ratio (9 patients)	6:3	
Phakic:Pseudophakic (n = 18 total eyes)	6:12	
History of vitrectomy	0	
Diagnoses (from incomplete medical histories)	No recorded eye disease	x5
	Glaucoma	x2
	Diabetes	x1
	Wet AMD	x1
TOD to cooling Mean ± SD (range)	6.3 ± 4.6 (1.6–14.6) hours	
TOD to enucleation Mean ± SD (range)	13.0 ± 4.8 (6.4–19.2) hours	
TOD to use Mean ± SD (range)	68.0 ± 30.2 (33.8–133.4) hours	

The surgical procedure simulated multiple needle ‘tap’ aspirations performed sequentially on the same eye. Each eye was passively warmed to room temperature and mounted in a support stand which held the eye and supported injection devices placed around it. Four 30G staked needle insulin syringes (Ultra-fine Insulin Syringe 12.7 mm x 30G x 0.5 mL, Becton Dickinson, Franklin Lakes, NJ) were inserted 3.5–4 mm from the limbus toward the center of the eye to a depth 6–8 mm, at arbitrary but equally spaced quadrants. Four prototype devices with 25G needles were inserted to a similar depth at alternating intervals between the staked needle syringes. After all needles were inserted in the eye and secured in the support stand, the plunger on the first prototype device was advanced to the aspirate position (Fig 1B). The vacuum sample container was observed for liquid filling for 5 s. The plunger was then advanced to the ‘drug delivery’ position to relieve the vacuum on the needle (Fig 1C)–however no fluid (or gas) was injected, as the drug cartridge was empty. The procedure was repeated for the remaining three prototype devices. After completing aspirations with the four prototype devices, aspiration was attempted with the staked needle 30G syringes. The plunger of each staked needle syringe was retracted to withdraw a maximum of 0.05 mL fluid. The operator released the plunger to relax the vacuum when either 0.05 mL was acquired, or 5 s had elapsed. After all eight aspirations were attempted all needles were withdrawn from the eye. Each needle was observed to watch for ‘wicking’ of the vitreous, forming a ‘string’ between the tip of the needle and the eye on withdrawal [8]. The final mass for each prototype was again measured on the precision balance.

The liquid biopsies were transferred from each acquisition device to a 0.5 mL conical polypropylene cryovial. Each vial was first labeled and weighed empty. The fluid samples contained in each staked needle syringe were expressed directly into a vial by pushing on the syringe plunger and extruding the sample back though the 30G needle. The sample was extracted from each prototype device by inserting a pair of 27G needles through ports on the prototype device into the sample collection chamber and pushing air into the sample chamber to displace the acquired sample into the cryovial. The final mass of each cryovial was again weighed. The samples were then frozen at -80 ºC until ready for analysis.

The precise volume of vitreous biopsy acquired by the prototype devices was determined as the difference between the mass of the devices at time of construction and after the surgical simulation, which was available for the final 60 (of 72) prototype devices and was used to calculate the variability of the volume of the vitreous biopsy acquired. The volume of vitreous biopsy extracted from the devices was determined as the difference in mass between the empty cryovials and those containing extracted samples. The volume of the biopsy collected by the staked needle syringes was determined simply as the mass extracted, as this process was extremely efficient in these low dead volume syringes. The density of vitreous was approximated as 1.00 g/mL [9]. An extracted biopsy volume greater than 25 μL was evaluated as successful.

A single liquid biopsy from each eye collected with the VitreoDx was analyzed for protein concentration at an offsite laboratory. If more than one sample met the laboratory’s volume criteria with an extracted volume in excess of 25 μL, the first sample aspirated from the eye was used. After the series of 18 eyes was completed, all samples were shipped on dry ice for offsite analysis with Proximity Extension Assay (Olink Proteomics Inc, Waltham MA). Target 96 Immuno-Oncology and Target 96 Oncology II panels were used on all submitted samples.

## Results

**Fig 2:**
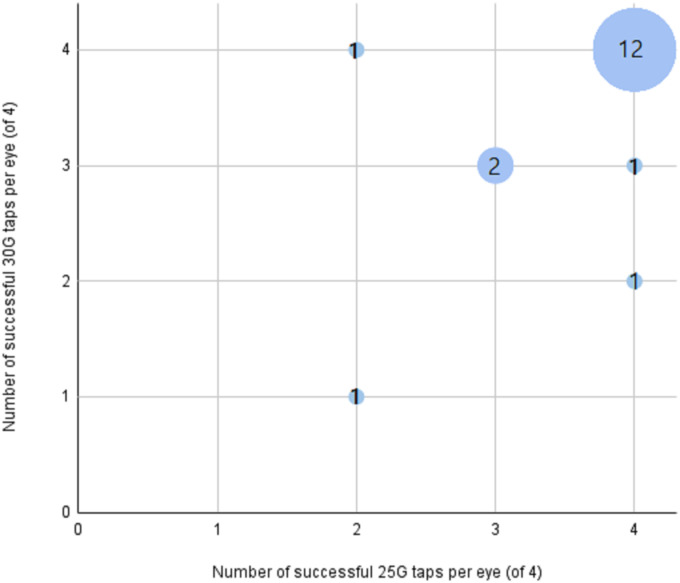
Comparison of tap success frequency by needle type per eye. The number of eyes with the same success frequency is indicated both by bubble size and number within the bubble. For 12 of the 18 eyes, all taps were successful for both 25G and 30G needles.

A sample usable for proteomic analysis was obtained from all 18 eyes using the prototype VitreoDx. Of the attempted liquid biopsies with the novel 25G VitreoDx, 92% (66 of 72) resulted in successful acquisition (>25 μL) while 89% (64 of 72) attempted by a traditional 30G needle resulted in a successful acquisition. All 16 failed aspirations were concentrated in 6 of the eyes. The number of eyes was insufficient to generate meaningful statistical conclusions, however the success rate with 25G and 30G in a particular eye appears to trend together (Fig 2) (Sheet ‘demographics and aspiration success’ in S1 Appendix). No trend between success rate and paired eyes of an individual, subject age, phakic status or other ophthalmic history, time to cooling, time to enucleation, time to use, or the order of aspiration was observed. A large fraction of the attempted acquisitions yielded a full sample and total liquid volume removed from each eye ranged from 231.2–493.6 μL (Table 2) (Sheet ‘biopsy volume’ in S1 Appendix). After biopsies were performed, all eyes were markedly soft to the touch. Vitreous wicking was not observed as any of the needles was retracted from the eyes.

**Table 2:** Sample acquisition.

Total number of 25G taps	72 (4x18 eyes)
Volume acquired Mean ± SD (range)	55.1 ± 14.6 (0.0–70.0) μL
Number of samples >25 μL	66
Total number of 30G taps	72 (4x18 eyes)
Volume acquired Mean ± SD (range)	50.9 ± 16.5 (0.9–87.7) μL
Number of samples >25 μL	64
Total liquid volume acquired from each eye Mean ± SD (range)	427.3 ± 63.6 (231.2–493.6) μL

**Fig 3:**
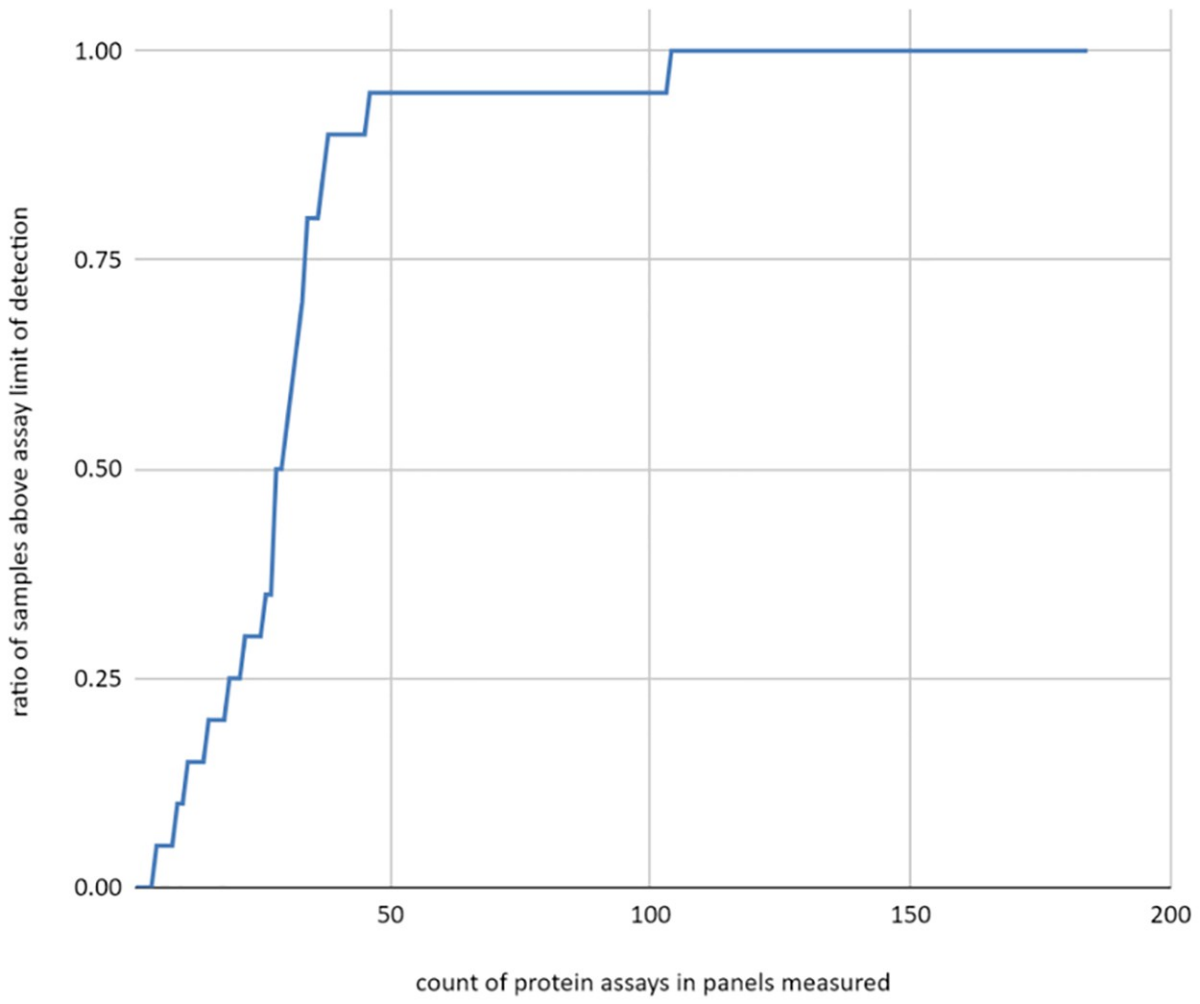
Detectability of proteins. Curve indicating the fraction of eyes that was measured above the level of detection in each protein assay.

Data returned from proteomic analysis was pre-analyzed from raw values and normalized for variation using internal and external controls. Protein concentration was expressed as normalized protein expression units (NPX), an arbitrary logarithmic unit which allows for relative quantification of protein concentration within a particular assay, *i*.*e*. across eyes within a single protein. The report also identifies the Limit of Detection (LOD) for each assay. (Sheet ‘Protein Concentration’ in S1 Appendix) All samples with an NPX > LOD are described as detected. Approximately 90% of the proteins assayed in the panels are detected in at least 25% of the collected samples (Table 3) and a majority of the proteins assayed are detected for almost all samples (Fig 3). For most assays performed, a trend of similar protein concentration was observed between paired eyes, and increasing protein concentration was observed with increasing duration between TOD and use.

**Table 3:** Protein assays.

Number of assays performed	184
Number of unique proteins assayed	166
Number of assays with at least 25% samples above LOD	164
Number of unique assays with at least 25% samples above LOD	149

**Fig 4:**
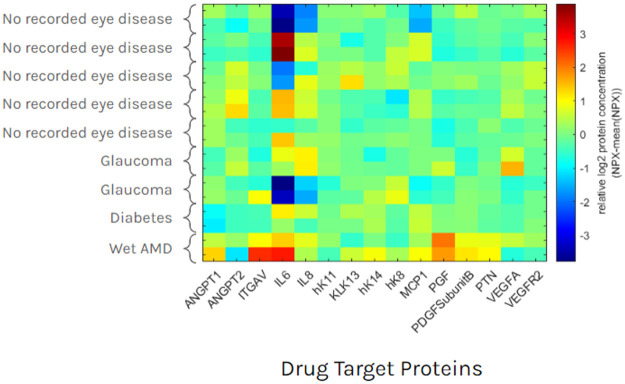
Relative concentration of examined drug targets. Differences in protein concentration are illustrative of protein concentration measured across the panels measured.

The relative concentration of measured proteins for a number of current and pipelined drug targets for neovascular age related macular degeneration (nvAMD) were isolated from the panel analysis (Fig 4). These data are illustrative of the variation seen across the panel. Some proteins, such as the kallikreins (hK11, KLK13, hK14, hK8) were observed with strongly detectable signals but had variation constrained within a factor of 2 across the 18 eyes. Other proteins, such as interleukin-6 (IL6) covered a more than a 100-fold range.

## Discussion

In the present study, we used an *ex vivo* model to explore the feasibility of routine vitreous biopsy, incidental to normally indicated intravitreal injection, with a novel prototype aspiration and injection device, VitreoDx. The key advantage of the device over traditional injection is the ability to acquire a liquid biopsy directly prior to injection using the same surgical needle insertion and adding only seconds to the in-eye procedure time. Our study primarily demonstrated that the prototype device was capable of reliably acquiring a liquid biopsy from human eyes, and that those samples could be used to quantify the relative concentrations of proteins of interest in vitreous fluid. The study also indicated that in eyes with significant age-related liquefaction, where a 25G needle was expected to acquire a biopsy with 90% success; it was also possible to acquire a biopsy with a much smaller needle with similar success. This has only been explored previously in smaller studies or in substantially different clinical indications [10, 11].

The eyes used in this study were an appropriate preclinical test for the biopsy function of our early stage VitreoDx prototype. The absence of a cutter in our device requires an eye with a degree of vitreous liquefaction. Unfortunately, no documented animal model provides a good representation of age-related vitreous liquefaction. Patients aged older than 75 years of age are commonly injected for nvAMD and previous clinical work indicates that 25G needle tap should be highly successful in this group. Eyes of this age are also typically ineligible as cornea transplant donors and therefore are more easily and ethically sourced as whole eyes [12]. We sourced the eyes to be as fresh as possible without working internal to an eye bank. Hours elapsed after death, as well as shipping conditions, (whether in saline or frozen) will contribute to some degree of physical and chemical changes relative to living eyes. These changes may include viscosity changes and increased or decreased levels of measurable proteins or ability to acquire a sample [13]. We note with encouragement, however, that vitreous humor is well known in forensic science for its low baseline metabolism, slow rate of decay, and remarkable ability to remain sterile for days after death [14]. It is further encouraging that the tap success rate for 25G needles closely matched the expectation set in the literature and did not show evidence of being significantly more aspirable. Comparison of the protein concentration relative to living eyes is complicated by enormous variability in protein concentration between individuals and the fact that the test method used is a relative quantitative measure within samples of the same run. We can compare the number of samples which measure within the measurable range of the tests of same technology. Lamy *et al*. reported that 80% of assays in their PEA panels yielded detectable results in at least 25% of surgically acquired samples [15]. We find that 90% of assays (166 of 184 assays including duplicate assays) within our PEA panels yielded detectable results in at least 25% of our *ex vivo* samples. We chose not to use the *ex vivo* human eye model to demonstrate the drug delivery portion of the VitreoDx in order to maximize the number of neat aspiration trials we could perform on each eye. Our previous unpublished work with the device in a variety of other gel phantoms suggested that the aspiration mode was most subject to variability due to the rheology of the phantom.

Our biopsy volume success criterion was oriented towards analysis. Our analysis vendor accepted a minimum fluid volume of 25 μL /sample, primarily to ease sample handling and minimize artifacts which may be generated due to sample/air interface issues. On the other hand, in clinic, the precise volume of the biopsy relative to the drug dose delivered is important either to minimize post injection IOP spike or avoid hypotony. In most cases, our device aspirated fluid until the chamber was filled to capacity. A much smaller minority of samples were partially acquired due to a blockage of the needle partway through the aspiration. Variability in the biopsy size collected with our prototype near the nominal aspirated volume was primarily driven by a known ±10% variation in length of the manually cut tubing of the vacuum sample chamber, rather than differences in vacuum maintenance or differences in the rheology of the vitreous. Greater manufacturing precision in the future will allow us to achieve better biopsy size repeatability and to explore these other important variables in more depth. Variability in the biopsy size collected with off-the-shelf syringes near the nominal aspirated volume of were driven largely by the ability of the operator to visualize the targeted volume and actuate the syringe.

Our protein analysis illustrates the application of analyzing individual biopsies for a large number of proteins. This technology and specific panels have previously been used to demonstrate differences in surgically extracted vitreous humor from proliferative diabetic retinopathy and non-diabetic controls. This study used a success criteria describing the number of samples measured within the dynamic range of the analysis method used. The test was not designed to measure the magnitude of any change introduced by the device on the protein measurements. We did not analyze a negative control sample to be aspirated through the device or measure the difference between samples collected with our prototype device and those collected with off-the-shelf syringes. We minimized the amount of time that biopsies sat in the prototype devices. Our previous experience with vitreous phantom solutions and an inflammatory protein panel indicated that interference from the custom sample tube was very low for most proteins for periods up to two hours at room temperature (unpublished).

The potential for use of a 30G needle removes a barrier to vitreous liquid biopsy in patients scheduled to receive a normally indicated intravitreal injection. The apparent correlation between ability to aspirate with 25G and 30G is important. Our finding suggests that in cases where the vitreous is liquified enough to be aspirable by a 25G needle, it is also likely aspirable by a 30G needle. Most doctors prefer to use a 30G needle for standard anti-VEGF injections [16, 17], and use larger needles if the injected formulation of the drug requires it due to viscosity or particle size [18]. Doctors describe a significantly higher penetration force with larger needles. The rate of reflux, which allows injected drug to escape the eye has been noted to be higher with larger needle diameter [19]. We look forward to a broader validation of this finding in live eyes, which might be performed immediately using off the shelf needles as a replacement for the 25G needles used in previously published protocols.

Although cartridge-based injectors have been used widely in medicine, primarily for dental anesthesia, patient self-injection, and emergency applications, the application to intravitreal injection appears to be novel and requires future validation [20]. Potential interactions between the biopsy and the drug delivery mode also warrant discussion. One interesting potential interaction involves the possibility of vitreous that may become caught in and block the primary needle, preventing drug delivery or creating tension on the retina. Firstly, this method relies on ‘liquid biopsy’. The similar performance of the 30G and 25G needles underscores the idea that formed vitreous, if present, is not well aspirated by needles of either gauge. Moving to 30G, because of its small cross-sectional area, will further ensure that formed vitreous cannot be aspirated [11]. Secondly, any vitreous strand that extends interiorly from the double-ended needle is very likely to be sheared when the sharp needle pushes through the tough septum of the drug cartridge, removing any vitreous ‘anchor’ on the interior of the device. When the plunger on the drug cartridge is depressed, the pressure difference between the syringe interior and the eye easily exceeds the original pressure difference between the eye and the evacuated sample container, and should displace any lodged vitreous in a reverse direction flush of the drug out of the needle. Another interesting potential interaction involves the possibility of contaminating the sample with the drug, which could interfere with some assays [21]. The currently investigated architecture is careful to ensure that no drug is released in the eye until the biopsy is acquired and isolated. Further, septa provided by the sample container and the drug cartridge work in series to prevent drug transfer from the cartridge into the sample along the exterior surface of the needle. To verify the proposed mitigations to rare or subtle phenomena will require experimental design to manufacture conditions most likely to generate the potential failures and sensitive failure detection, preferably without introducing the variability inherent to human tissue. Ultimately, both the biopsy and drug delivery functions of a production equivalent device will require validation in live eyes of relevant human subjects.

Vitreous liquid biopsy has potential to address multiple emergent issues facing intravitreal retina care. The removal of fluid volume prior to injection prophylactically manages intraocular pressure (IOP) spike [22]. This need is growing in relevance, as recently approved drugs for geographic atrophy are formulated with volumes near the maximum of tolerability without paracentesis, and the requirement to concurrently treat nvAMD exacerbates the problem [23, 24]. Reduction in post injection IOP may also reduce the incidence of vitreous reflux and therefore keep more of the injected drug in the eye [25]. Analysis of the biopsy has potential to guide follow up care. An analysis panel consisting of the concentration of drug [26], drug target [27], downstream proteins [28], proteins in parallel disease processes [29], anti-drug antibodies [30], or other analytes may provide clinicians with an useful supplement to structural imaging as a biomarker to help select appropriate therapy and adjust dose timing [31]. Within research settings, analysis of a liquid biopsy has potential to increase the probability of clinical trial success [32] and identify targets for new therapeutics [33]. VitreoDx, in combination with powerful protein assay technology, can yield data that might be used first to enrich a clinical trial with subjects who express a novel drug target in high ratio relative to traditional targets, might one day guide patient selection as a companion diagnostic (Fig 4). It is our hope that the innovation demonstrated in VitreoDx reduces clinical barriers to routine vitreous liquid biopsy so that this procedure can deliver the full promise of its potential for retinal care.

## Supporting information

S1 Appendix Supplemental data. Sheet ‘demographics and aspiration success’ describes each eye used in testing and the rate of aspiration success with each needle type. Sheet ‘biopsy volume’ describes the volume acquired from each needle. Sheet ‘Protein concentration’ provides the raw NPX values for each eye, and each protein assay within the two proximity extension assay panels Olink Target 96 Immuno-Oncology(v.3112) and Olink Target 96 Oncology II(v.7005).
(XLSX)
